# Stratify severe risk in children with respiratory syncytial virus pneumonia—A retrospective study based on machine learning and SHAP interpretation

**DOI:** 10.3389/fped.2026.1775752

**Published:** 2026-03-16

**Authors:** Jun-An Pan, Wen-Hao Yang, Chao-Fen Wu, Min Zhou, Juan Liu, Li-Na Chen

**Affiliations:** 1 Department of Pediatric Pulmonology, West China Second University Hospital, Sichuan University, Chengdu, China; 2 Key Laboratory of Birth Defects and Related Diseases of Women and Children (Sichuan University), Ministry of Education, Chengdu, China; 3 NHC Key Laboratory of Chronobiology (Sichuan University), Chengdu, China

**Keywords:** children, machine learning, pneumonia, respiratory syncytial virus (RSV), shapley additive explanations (SHAP)

## Abstract

**Background:**

Respiratory syncytial virus (RSV) is the primary pathogen causing severe lower respiratory tract infections in children, imposing a significant disease burden worldwide. The clinical manifestation of respiratory syncytial virus is not highly specific, in severe cases, it may cause a severe inflammatory response in the organism, potentially resulting in mortality. Currently, early identification and risk stratification tools for severe RSV-related pneumonia remain inadequate. This study identified potential high-risk factors contributing to severe cases in children with respiratory syncytial virus pneumonia by screening variables and establishing machine learning models, aiming to achieve individualized prevention, diagnosis, and treatment for these patients.

**Methods:**

Our study conducted variable screening through univariate analysis and multivariate logistic regression analysis. The performance of five machine learning models in the training and test sets was compared using receiver operating characteristic curves, and the XGBOOST model with the best overall performance was selected as the final model. Finally, shapley additive explanations (SHAP) was employed to quantify and perform clinically interpretable analysis on this black-box model.

**Results:**

Twelve key variables were identified in patients with severe respiratory syncytial virus pneumonia. XGBoost demonstrated the best overall performance, selected as the final model for the study, which achieving AUC values of 0.949 and 0.818 in the training and test sets respectively. By SHapley Additive exPlanations (SHAP), it was found that fever duration, diarrhea, hemoglobin concentration, rhinorrhea, age, neutrophil-to-lymphocyte ratio, gestational age, neutrophil count, mode of delivery, and lymphocyte count may be the most important predictive variables for children with severe RSV pneumonia.

**Conclusion:**

Our findings demonstrated that prolonged fever duration, presence of diarrhea, decreased hemoglobin concentration (HGB), absence of rhinorrhea, age under 3 months (Age<3 m), and elevated neutrophil-to-lymphocyte ratio (NLR) were predictors of severe cases among children with RSV pneumonia.

## 1 Introduction

Respiratory syncytial virus (RSV) has imposed a substantial disease burden prominently among patients younger than 5 years, as the primary pathogen of lower respiratory tract infections in children worldwide (1). It is estimated that RSV infections accounts for approximately 3.6 million hospitalizations and over 100,000 deaths among children under 5 years old in 2019 (1). Multiple studies have shown that hospitalization cases of RSV infection are concentrated in infants under 1 year of age, with severe RSV pneumonia predominantly observed in infants younger than 3 months (2–4). In real-world data from Italy, the direct medical costs associated with RSV-related hospitalizations were significantly higher than those in the general population, with the majority of cases being healthy full-term infants (5). Therefore, accurately identifying the risk factors for RSV pneumonia is of vital importance for early intervention, risk stratification, and clinical decision-making (6). Moreover, sustained interdisciplinary collaboration, localized evidence generation, and community engagement will be essential for achieving comprehensive management of RSV (7).

Currently, there are extensive research on risk factors for severe RSV infection. For example, multiple studies have found that prolonged duration of fever is associated with the severity of RSV pneumonia, due to high risk of complications, bacterial pneumonia and prolonged hospital stay (8–10). It is confirmed that age as a key risk factor for severe RSV infection, which infants ≤3 months old had a significantly increased risk of requiring passive oxygen therapy and ICU admission among RSV-positive children was significantly associated with younger age (11, 12). Although previous studies have explored the risk factors for severe RSV pneumonia, they commonly employ univariate analysis and traditional regression models as statistical methods, which have limitations in interpreting the relationships between complex characteristic variables (13).

Machine learning-based methods, such as XGBoost models, demonstrate significant advantages in discovering potential connections between variables (14, 15). So that, machine learning-based models seem to be capable of early identification and risk stratification of severe RSV pneumonia in children. We created a XGBoost model and conduct a SHAP interpretability analysis on this black-box model, using the data collected from a local hospital system.

In this study, our primary objective was to identify the risk factors for severe RSV pneumonia in children. Also, we analyzed the interrelationships among these risk factors, and quantified the magnitude of their impact on the development of severe RSV pneumonia in children. Furthermore, We aimed to provide evidence-based strategies for precise and individualized prevention and treatment of severe RSV pneumonia.

## 2 Method

### 2.1 Research subject

In this retrospective study, we collected and analyzed clinical data from pediatric patients hospitalized with RSV pneumonia at West China Second University Hospital of Sichuan University between January 1, 2019 and February 29, 2024. These patients constituted a cohort of pediatric inpatients admitted to the respiratory ward, all with a confirmed diagnosis of RSV pneumonia. This study was approved by the Ethics Committee of West China Second University Hospital of Sichuan University, with the ethical approval number: Medical Research 2022 Ethical Approval No. (074).

### 2.2 Inclusion and exclusion criteria

Patients were included if they met all of the following criteria: (1) diagnosed with pneumonia, meeting the criteria of the 2019 edition of the “Diagnosis and Treatment Guidelines for Children with Community-Acquired Pneumonia” (16); (2) age ≤14 years old; (3) positive nucleic acid or antigen test for RSV. The exclusion criteria were as follows: (1) nosocomial infection; (2) hematologic malignancies; (3) participants in drug or medical device clinical trials; (4) patients with incomplete clinical data.

### 2.3 Diagnostic criteria for severe cases

Meeting any one criterion is sufficient, referring to the “Diagnosis and Treatment Guidelines for Community-Acquired Pneumonia in Children (2019 Edition)” as follows: (1) deterioration of general condition: disturbance of consciousness, feeding difficulties, or significant dehydration; (2) insufficient work of breathing: oxygenation disorder (SpO₂<92% or cyanosis), dyspnea with abnormal rate (infants ≥70 bpm, children over 1 year old ≥50 bpm), increased work of breathing (nasal flaring, positive three depression sign, groaning) or apnea; (3) fever condition: ultra high grade fever, or persistent high grade fever >5 days; (4) chest imaging examination: extensive consolidation involving two-thirds or more of a unilateral lung, multilobar consolidation, pleural effusion, pneumothorax, atelectasis, pulmonary necrosis, and lung abscess; (5) extra-pulmonary complications (16).

### 2.4 Data collection

The collected data included: (1) demographics: age, sex, season of onset (the season coinciding with the time of diagnosis), birth weight, gestational age, parity, mode of delivery, and feeding method; (2) clinical manifestations: fever, fever period (days of recurrent fever), wheezing, sputum production, rhinorrhea, wheezing, emesis, and diarrhea; (3) laboratory tests: complete blood count parameters (collecting the first blood test since onset, typically within 1 week of onset): white blood cell count (WBC), hemoglobin (HGB), platelet count (PLT), neutrophil count (ANC), lymphocyte count (ALC), C- reactive protein (CRP), neutrophil-to-lymphocyte ratio (NLR); (4) others: co-infection.

### 2.5 RSV etiological detection methods

For the etiological detection of RSV, the specimen types collected are nasopharyngeal swabs, sputum, and bronchoalveolar lavage fluid, which are tested using antigen detection or nucleic acid detection methods.

Antigen testing: Detect RSV antigens in respiratory tract samples. The testing was performed using the 7-respiratory virus detection kit (immunofluorescence method) from Diagnostic Hybrids, Inc., USA. This assay is based on the principle of antigen-antibody specific binding, with the kit containing fluorescein-labeled specific monoclonal antibodies against RSV, influenza A virus, influenza B virus, adenovirus, and parainfluenza virus types 1, 2, and 3. Specialized nurses collected nasopharyngeal swab samples from pediatric patients, placing the swabs into sampling tubes containing buffer solution. After sample collection, the tubes were vortexed, and the supernatant was taken. Sample processing solution and fluorescein-labeled antibody mixture were added to the reaction plate wells, followed by incubation at 37 °C for a specified duration. After 3–5 washes, mounting medium was added, and results were interpreted under a fluorescence microscope. If distinct specific fluorescence appeared in the sample well with intensity meeting or exceeding the preset positive threshold, the sample was determined to be RSV antigen-positive.
Nucleic Acid Testing: 1) Multiplex Nucleic Acid Detection of Respiratory Pathogens: The 13 respiratory pathogen co-detection kit developed by Ningbo Health Gene Technologies Co., Ltd., based on Polymerase Chain Reaction (PCR)-capillary electrophoresis technology, was used to analyze respiratory samples collected from pediatric patients. After standardized sample collection by professional nursing Homo sapiens personnel, the samples were immediately preserved in a 15 mL sterile tube containing 2.5 mL of viral preservation solution. This testing system integrates multiplex reverse transcription PCR amplification with capillary electrophoresis analysis, enabling simultaneous detection of nucleic acids from multiple common respiratory pathogens, including RSV, Homo sapiens bocavirus, Mycoplasma pneumoniae, influenza A/B viruses, adenovirus, rhinovirus, parainfluenza virus, coronavirus, Homo sapiens metapneumovirus, and Chlamydia. 2) Seven Respiratory Pathogen RNA Detection: The seven respiratory pathogen nucleic acid detection kit (dual amplification method) from Wuhan Zhongzhi Biotechnology Co., Ltd. was employed for testing. Utilizing a unique dual amplification technique (DAT), which combines RNA isothermal amplification and multi-biotin signal amplification, the test detects nucleic acids of RSV, influenza A virus, influenza B virus, parainfluenza virus, adenovirus, Mycoplasma pneumoniae, and Chlamydia pneumoniae.

### 2.6 Statistical analysis

#### 2.6.1 Descriptive statistical analysis

The outcome variables were set as mild and severe cases. Descriptive statistical analysis was performed on the dataset. Chi-square tests were used for categorical variables. The Shapiro–Wilk test (abbreviated as the S-W test) was employed to assess the normality of continuous variables. Since none of the continuous variables in the dataset followed a normal distribution, the Wilcoxon rank-sum test (Mann–Whitney *U*-test) was applied for statistical analysis of continuous variables. A *p* value <0.05 was considered significant in analysis.

#### 2.6.2 Model establishment and comparison

The dataset was divided into an 80%: 20% ratio for the training set and test set. First, we seleced variables with statistical significance (*p* value < 0.05), the season of onset and age as initial feature variables, considering statistical and RSV epidemiological characteristics. Second, the glm function was used to establish an initial multivariate logistic regression model. Subsequently, stepwise variable selection was performed to identify the final variables included in the model training. Finally, we elected five machine learning models (logistic regression, random forest, support vector machine, XGBoost, and naive Bayes) using the selected variables, and conducted model comparisons using ROC curves in both the training and test sets. The XGBoost model demonstrating the best performance in both the training and test sets (AUROC = 0.949 in the training set, AUROC = 0.818 in the test set).

To improve XGBoost performance and reduce overfitting, this study used grid search to optimize hyperparameters (e.g., learning rate, max depth, subsample ratio) via 10-fold cross-validation, maximizing AUC to find the best settings. L1 regularization was added to limit complexity, and early stopping was set to 200 iterations with a 50-round patience for no improvement. The final model was trained on the full dataset with these optimal parameters. A fixed random seed ensured reproducibility.

#### 2.6.3 SHAP interpretation of the XGBoost model

The Fastshap package was employed to perform SHAP interpretation on the XGBoost model. The analysis focused on both global and local interpretability. On one hand, for a global perspective, we generated a summary plot to rank features by their mean absolute SHAP value, and created a beeswarm plot to illustrate the relationship between the value of a feature and its impact on the model's output across the entire dataset. On the other hand, for instance-level explanations, We used dependency graphs to interpret the contribution of individual features and employed force diagrams to demonstrate how feature variables achieve the final prediction.

#### 2.6.4 Data processing and statistical software

EXCEL was used for raw data recording and conversion into CSV format files. Data format adjustment, variable screening, statistical analysis, machine model construction, and SHAP interpretation were all completed in R4.5.1 software.

## 3 Result

### 3.1 Descriptive statistical analysis of baseline characteristics

The baseline characteristics of all 1,269 RSV pneumonia patients are described in Table 1, including 836 severe RSV pneumonia cases, accounting for 65.9%. Among them, statistically significant demographic variables included gestational age (*P* < 0.001), mode of delivery (*P* = 0.015), birth weight (*P* = 0.009), and parity (*P* = 0.007). Clinical manifestation variables comprised fever (*P* < 0.001), fever period (*P* < 0.001), rhinorrhea (*P* < 0.001), wheezing (*P* = 0.045), emesis (*P* = 0.003), and diarrhea (*P* < 0.001). Laboratory test variables consisted of absolute neutrophil count (ANC, *P* < 0.001), absolute lymphocyte count (ALC, *P* < 0.001), neutrophil-to-lymphocyte ratio (NLR, *P* < 0.001), C-reactive protein (CRP, *P* < 0.001), and hemoglobin level (HGB, *P* < 0.001).

**Table 1:** Descriptive statistical analysis of baseline characteristics for all patients.

Variable	All patients, *N* (%)	Severe cases, *N* (%)	*p*. overall
	*N* = 1,269 (100%)	*N* = 836 (65.9%)	
Sex:			0.829
Female	795 (62.6%)	526 (62.9%)	
Male	474 (37.4%)	310 (37.1%)	
Season:			0.370
Spring	231 (18.2%)	147 (17.6%)	
Summer	700 (55.2%)	456 (54.5%)	
Autumn	265 (20.9%)	179 (21.4%)	
Winter	73 (5.7%)	54 (6.5%)	
Gestational age:			<0.001*
Full-term	214 (16.9%)	163 (19.5%)	
Premature	1,055 (83.1%)	673 (80.5%)	
Mode of delivery:			0.015*
Eutocia	380 (29.9%)	231 (27.6%)	
Cesarean	889 (70.1%)	605 (72.4%)	
Feeding method:			0.756
Breastfeeding	563 (44.4%)	374 (44.7%)	
Others	706 (55.6%)	462 (55.3%)	
Birth weight:			0.009*
<2,500 g	191 (15.0%)	144 (17.2%)	
≥2,500 g–≤4,000 g	35 (2.8%)	21 (2.5%)	
>4,000 g	1,043 (82.2%)	671 (80.3%)	
Fever:			<0.001*
No	397 (31.3%)	227 (27.2%)	
Yes	872 (68.7%)	609 (72.8%)	
Expectoration:			0.938
No	1,011 (79.7)	665 (79.5%)	
Yes	258 (20.3%)	171 (20.5%)	
Rhinorrhea:			<0.001*
No	783 (61.7%)	548 (65.6%)	
Yes	486 (38.3%)	288 (34.4%)	
Wheezing:			0.045*
No	381 (30.0%)	235 (28.1%)	
Yes	888 (70.0%)	601 (71.9%)	
Emesis:			0.003*
No	1,009 (79.5%)	644 (79.4%)	
Yes	260 (20.5%)	192 (20.6%)	
Diarrhea:			<0.001*
No	852 (67.1%)	487 (58.3%)	
Yes	417 (32.9%)	349 (41.7%)	
Co-infection:			0.806
No	734 (57.8%)	481 (57.5%)	
Yes	535 (42.2%)	355 (42.5%)	
Age:			0.762
≥5y	44 (3.5%)	28 (3.4%)	
<3m	401 (31.6%)	271 (32.4%)	
3m–<6m	196 (15.4%)	124 (14.8%)	
6m–<1y	221 (17.4%)	152 (18.2%)	
1y–<2y	176 (13.9%)	113 (13.5%)	
2y–<5y	231 (18.2%)	148 (17.7%)	
Fever period, IQR(P25,P75)		3.00 (0.00;8.00)	<0.001*
CRP(mg/L), IQR(P25,P75)		0.80 (0.50;6.50)	<0.001*
Parity, IQR(P25,P75)		1.00 (1.00;2.00)	0.007*
HGB(g/L), IQR(P25,P75)		113 (102;123)	<0.001*
WBC(10^9^/L), IQR(P25,P75)		7.90 (5.80;10.40)	0.059
PLT(10^9^/L), IQR(P25,P75)		364 (277;458)	0.153
ANC(10^9^/L), IQR(P25,P75)		2.55 (1.48;4.18)	<0.001*
ALC(10^9^/L), IQR(P25,P75)		3.84 (2.43;5.53)	<0.001*
NLR, IQR(P25,P75)		0.64(0.33;1.44)	<0.001*

The symbol * indicates *P* < 0.05, which is considered statistically significant.

### 3.2 Variable screening and model comparison

**Figure 1:**
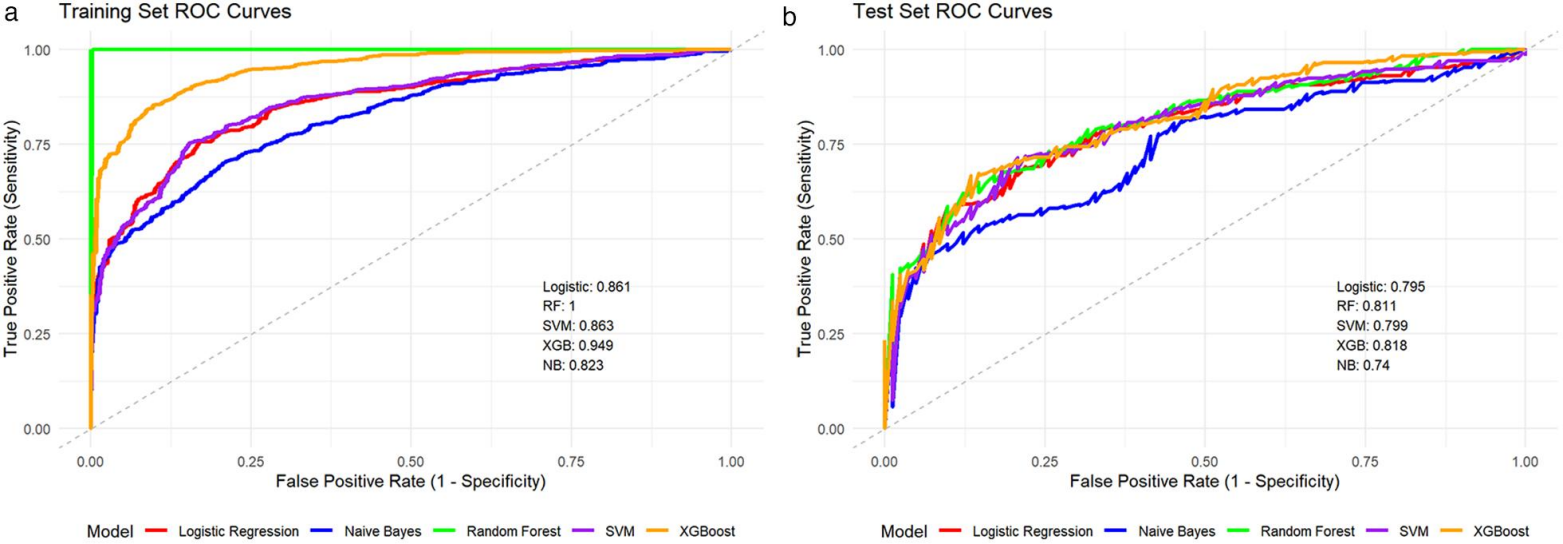
Comparison of receiver operating characteristic curves on the test set: **(a)** for training set and **(b)** for test set.

First, through univariate analysis, multivariate logistic regression analysis, and stepwise regression analysis, twelve key variables were identified (showed in Table 2): age, gestational age, mode of delivery, birth weight, wheezing, diarrhea, fever period, hemoglobin (HGB), absolute neutrophil count (ANC), absolute lymphocyte count (ALC), and neutrophil-to-lymphocyte ratio (NLR). Second, we established five models (logistic regression models, random forest models, support vector machine models, XGBoost models, and naive Bayes models) using these feature variables in both the training set and the test set. Figures 1a,b shows the receiver operating characteristic (ROC) curves of the five models in the training set and the test set. Ultimately XGBoost was selected as the final model for this study, which demonstrated the best overall performance, achieving AUC values of 0.949 and 0.818 in the training and test sets respectively. Furthermore, the random forest model was considered to exhibit over-fitting, as the AUC values on the training set and test set were 1.0 and 0.818 respectively.

**Table 2:** Multivariate logistic regression analysis in the training group.

Variable	Ref	crude OR (95%CI)	adj. OR (95%CI)	P (Wald's test)	P (LR-test)
Age	≥5y				<0.001*
<3m		0.95 (0.45,2.03)	3.69 (1.05,13.05)	0.042*	
3m–<6m		0.78 (0.36,1.71)	1.43 (0.4,5.13)	0.584	
6m–<1y		1.07 (0.49,2.35)	1.42 (0.4,5.05)	0.591	
1y–<2y		0.96 (0.43,2.12)	0.67 (0.19,2.37)	0.531	
2y–<5y		0.76 (0.35,1.66)	0.64 (0.19,2.19)	0.481	
Gestational age	Premature vs. Full-term	0.5 (0.34,0.73)	0.62 (0.35,1.11)	0.108	0.106
Mode of delivery	Cesarean vs. Eutocia	1.28 (0.97,1.69)	1.81 (1.26,2.61)	0.001*	0.001*
Birth weight	<2,500 g				0.015*
≥2,500 g–≤4,000 g		0.36 (0.16,0.84)	0.27 (0.09,0.84)	0.023*	
>4,000 g		0.47 (0.31,0.7)	0.42 (0.23,0.79)	0.007*	
Fever	Yes vs. No	1.72 (1.3,2.27)	0.38 (0.24,0.6)	<0.001*	<0.001*
Wheezing	Yes vs. No	1.32 (1,1.75)	1.66 (1.14,2.43)	0.008*	0.008*
Diarrhea	Yes vs. No	3.77 (2.73,5.22)	4.95 (3.3,7.4)	<0.001*	<0.001*
Fever period		1.33 (1.26,1.41)	1.83 (1.63,2.05)	<0.001*	<0.001*
HGB (g/L)		0.97 (0.96,0.98)	0.98 (0.97,0.99)	0.002*	0.002*
ANC (10^9^/L)		1.12 (1.05,1.19)	1.13 (1.02,1.24)	0.02*	0.014*
ALC (10^9^/L)		0.9 (0.86,0.95)	0.91 (0.85,0.97)	0.006*	0.005*
NLR		1.16 (1.04,1.29)	0.95 (0.83,1.09)	0.456	0.397

The symbol * indicates *P* < 0.05, which is considered statistically significant.

### 3.3 Using SHAP to visualize and interpret the XGBoost model

**Figure 2:**
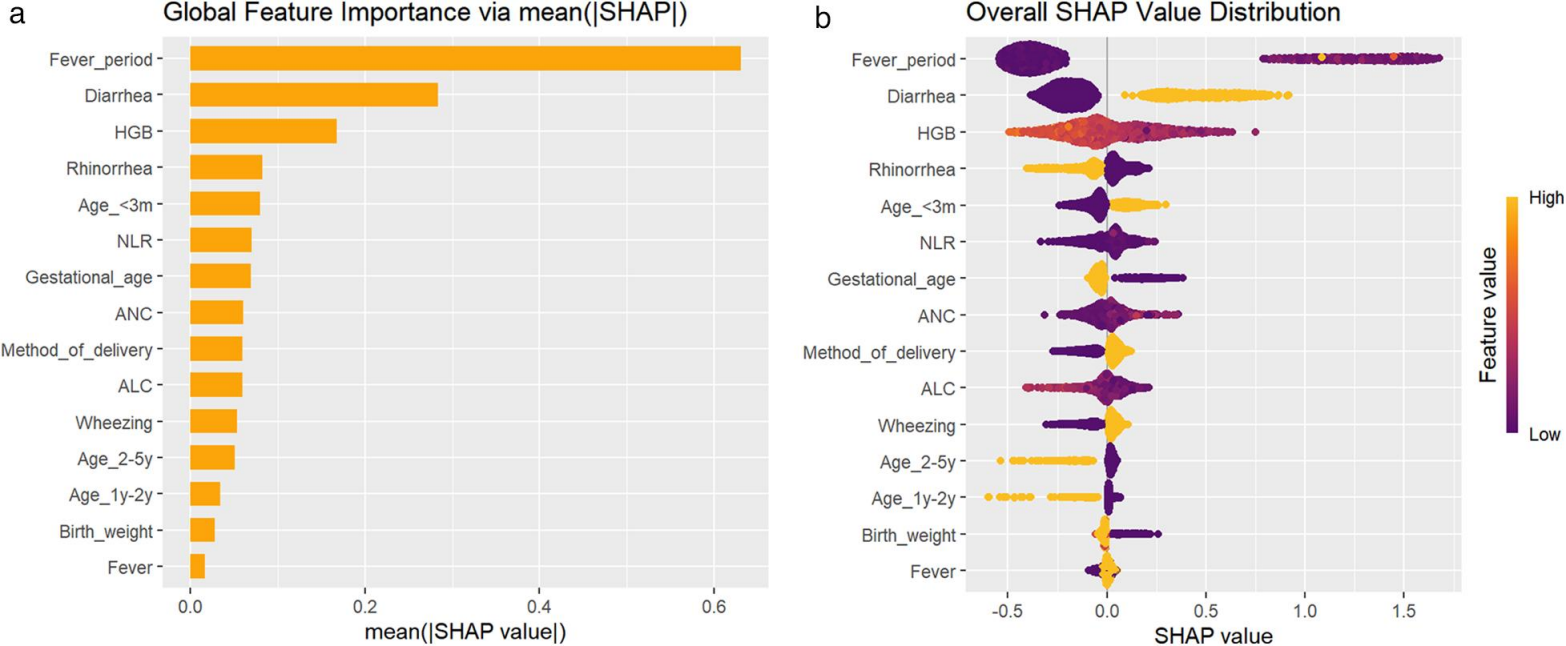
SHAP interpretation of overall characteristics for the XGBoost model. **(a)** Displays the ranking of feature importance based on the mean absolute SHAP values; **(b)** illustrates the distribution of SHAP values for each feature.

By calculating SHAP values, the contribution of each feature to the model's predictions is visually presented. Figure 2a displays the ranking of feature importance based on the mean absolute SHAP values. The top ten feature variables in order are fever duration, diarrhea, HGB, rhinorrhea, age less than 3 months, NLA, gestational age, ANC, mode of delivery, and ALC. The bee swarm plot in Figure 2b illustrates the distribution of SHAP values for each feature, where yellow dots represent positive SHAP values and purple dots indicate negative SHAP values.

**Figure 3:**
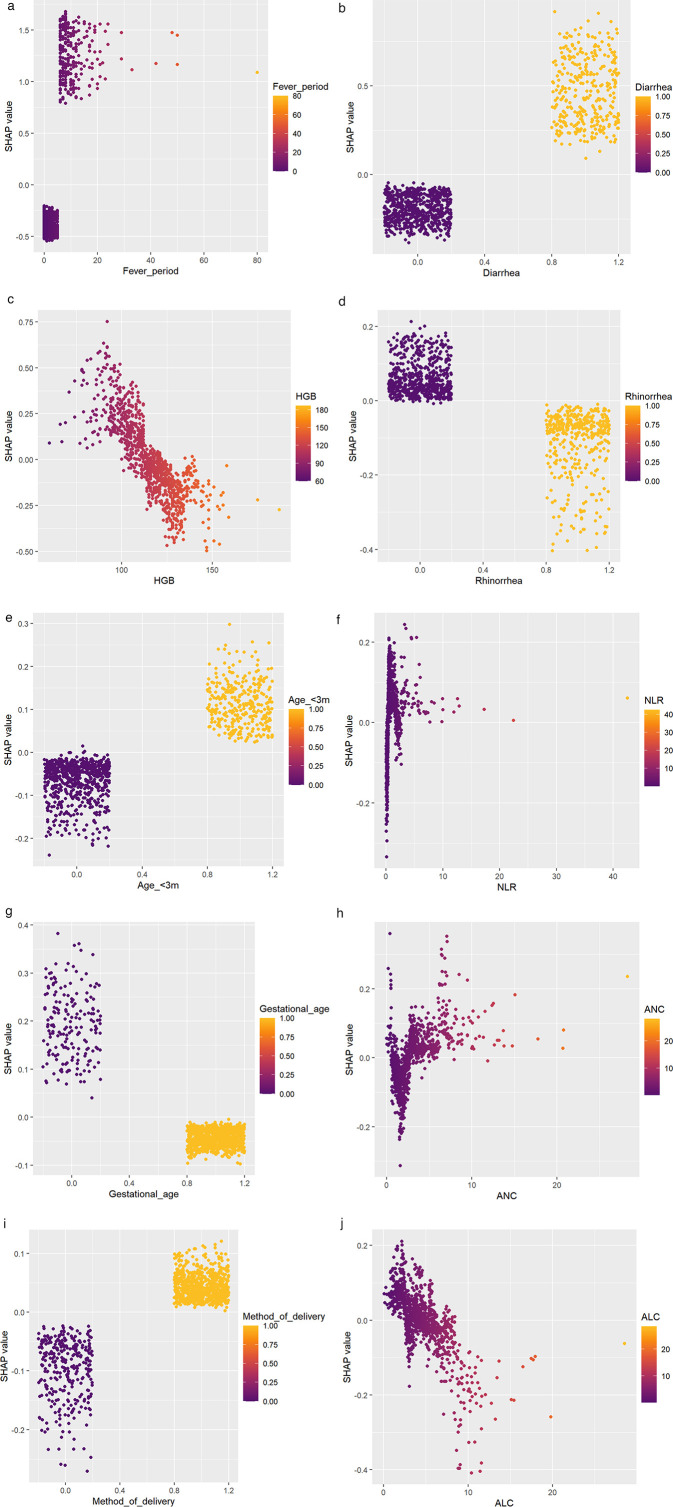
Dependency graph of the top 10 contributing individual features for the XGBoost model. **(a)** Demonstrates the correspondence between Shap values and fever period; **(b)** shows that the presence of diarrhea has a positive impact on the prediction outcome; **(c)** indicates that the Shap value increases as HGB decreases; **(d)** demonstrates that the presence of rhinorrhea has a negative effect on the prediction outcome; **(e)** reveals that an age of less than 3 months has a positive impact on the prediction outcome; **(f)** shows that the Shap value exhibits an upward trend as the NLR value increases; **(g)** illustrates that being a full-term infant has a positive effect on the prediction outcom; **(h)** indicates that the Shap value increases with the rise in ANC; **(i)** demonstrates that cesarean section has a positive impact on the prediction outcome; **(j)** shows that the Shap value decreases as the ALC increases.

**Figure 4:**
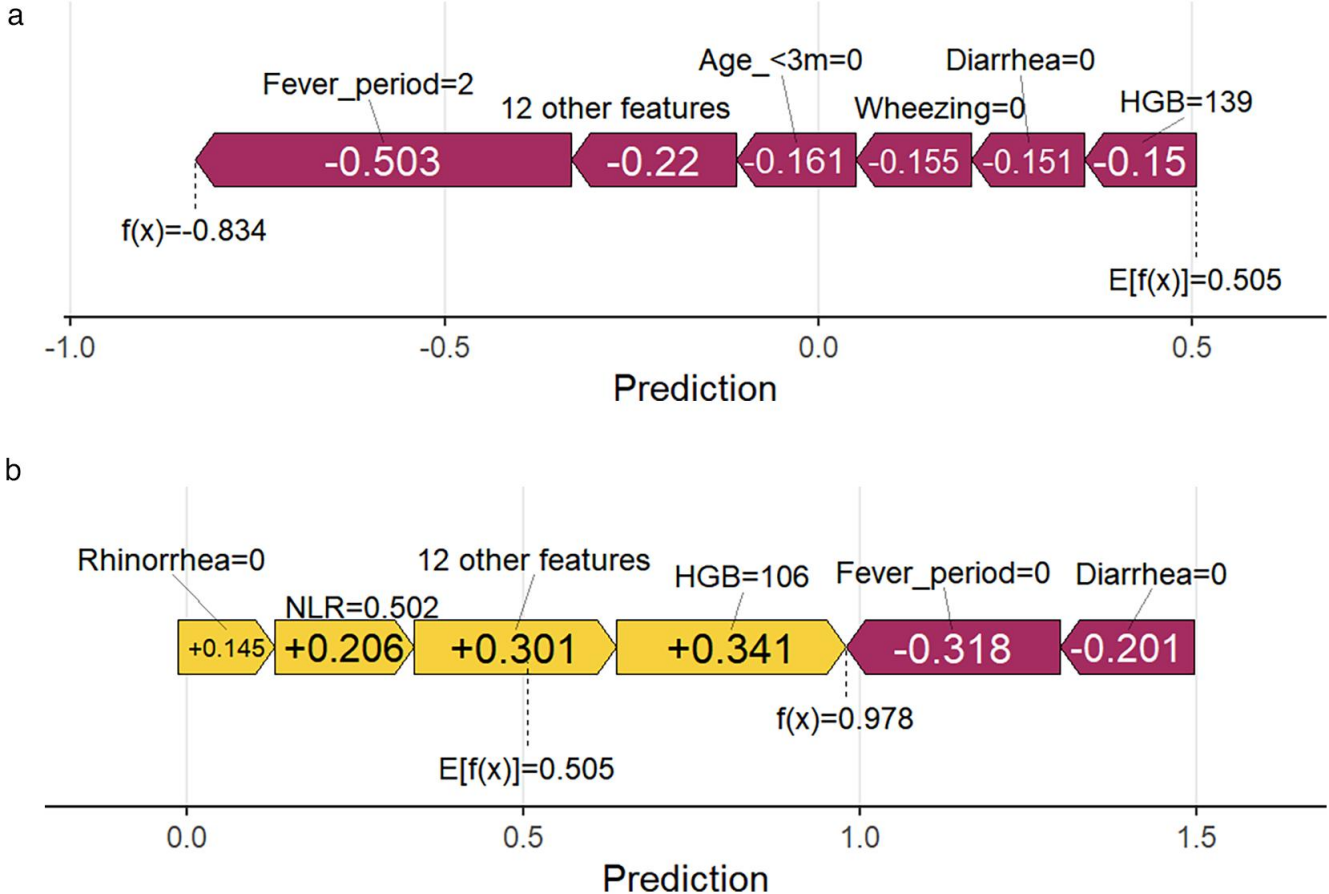
SHAP force plots for two representative patients: feature contributions to predicted outcomes. **(a)** A fever period of 2 days, not being younger than 3 months, absence of wheezing, absence of diarrhea, and higher HGB had a negative influence on the prediction outcome; **(b)** the absence of rhinorrhea, higher NLR, and lower HGB had a positive impact on the prediction outcome.

To explore the association between model features and diagnostic significance, we conducted a feature dependency analysis. Figure 3a demonstrates the correspondence between Shap values and fever period, showing an increase with prolonged fever days. A critical point emerged at approximately 5 days of fever period. Below this threshold, Shap values were predominantly negative, while above it they became mainly positive, which aligns with the general diagnostic criteria for severe pneumonia. Figure 3b shows that the presence of diarrhea has a positive impact on the prediction outcome, while the absence of diarrhea has a negative impact. Figure 3c indicates that the Shap value increases as HGB decreases, suggesting that a trend toward anemia during the disease course has a positive influence on the prediction outcome. Figure 3d demonstrates that the presence of rhinorrhea has a negative effect on the prediction outcome, while its absence has a positive effect. Figure 3e reveals that an age of less than 3 months has a positive impact on the prediction outcome. Figure 3f shows that the Shap value exhibits an upward trend as the NLR value increases, indicating that a higher NLR has a positive influence on the prediction outcome. Figure 3g illustrates that being a full-term infant has a positive effect on the prediction outcome, whereas being a preterm infant has a negative effect. Figure 3h indicates that the Shap value increases with the rise in ANC. Figure 3i demonstrates that cesarean section has a positive impact on the prediction outcome, while vaginal delivery has a negative impact. Figure 3j shows that the Shap value decreases as the ALC increases. Figures 4a,b present two typical cases (one mild RSV pneumonia patient and one severe RSV pneumonia patient). In Figure 4a, a fever period of 2 days, not being younger than 3 months, absence of wheezing, absence of diarrhea, and higher HGB had a negative influence on the prediction outcome. In Figure 4b, the absence of rhinorrhea, higher NLR, and lower HGB had a positive impact on the prediction outcome, while the absence of fever and diarrhea had a negative effect on the predicted prognosis.

## 4 Discussion

In this study, we conducted a retrospective descriptive analysis of 1,269 hospitalized children with respiratory syncytial virus pneumonia to identify demographic, clinical, and laboratory factors associated with disease severity. A combination of traditional univariate analysis and multivariate logistic regression analysis was employed, with clinical relevance meticulously evaluated to inform the selection of input variables for the machine learning model. Subsequently, we developed five machine learning models and compared their performance using receiver operating characteristic curves. Finally, SHAP-based interpretability analysis was applied to enhance both the performance assessment and transparency of the XGBoost model, which demonstrated superior predictive accuracy in both training set and test set. As an ensemble learning method that utilizes decision trees as base learners, XGBoost model is capable of effectively addressing high-dimensional data, nonlinear relationships, variable interactions, and multicollinearity, thereby offering novel analytical perspectives and facilitating the discovery of insights within this study. The results of this study indicate that fever period, diarrhea, HGB, rhinorrhea, age, NLR, gestational age, ANC, mode of delivery, and ALC are the most important predictive variables. These findings will inform future efforts to provide a feasible and practical tool for the early identification of children at potential risk of severe RSV pneumonia, facilitating more precise risk stratification and individualized clinical interventions.

The findings of this study are consistent with current researches, that prolonged fever period, anemia and infants under 3 months of age are more prone to developing severe RSV infection. Although previous studies have shown that the prolonged fever period has predictive value for the prognosis of RSV-related illnesses, which indicates high viral load and intense T-cell response (17, 18), our findings demonstrates a strong correlation between fever duration and the occurrence of severe RSV pneumonia. RSV infection induces a Th2-type immune response, promoting the secretion of IL-4 and IL-10, which may affect iron metabolism and hemoglobin synthesis (19). This in turn reduces blood oxygen-carrying capacity, exacerbating the hypoxemia caused by RSV (20). As some studies have shown that infants aged < 3 months (Age<3 m) have the highest incidence of severe RSV infection, we similarly identified age under 3 months as an independent significant risk factor for severe disease progression in pediatric patients with RSV pneumonia (21, 22).

In our study, full-term infants and children delivered via cesarean section are more prone to developing severe cases when afflicted with RSV pneumonia. It is noteworthy that although preterm infants with underlying congenital conditions are at high risk of severe infections, existing research data indicate that the majority of children with severe RSV pneumonia are full-term infants (23, 24). Compared to preterm infants, full-term infants may face higher collective exposure risks to RSV infection and exhibit more intense inflammatory responses under RSV infection. This may be a reasonable explanation. Cesarean section increases the risk of severe infections, which may be attributed to the fact that maternal microbial colonization during vaginal delivery promotes the maturation of the neonatal immune system, while cesarean section delays the establishment of Th1/Th2 balance (25, 26).

Our study quantified the predictive value of neutrophil-to-lymphocyte ratio (NLR) and diarrhea in predicting severe RSV infection, as few studies have done this before. The neutrophil-to-lymphocyte ratio (NLR), as an easily accessible inflammatory marker, has shown significant value in recent years for assessing the severity of infectious diseases (27). Elevated NLR typically reflects enhanced inflammatory responses and immune dysregulation, which indicates neutrophil mobilization and lymphocyte depletion (28, 29). Considering that the NLR is readily available in early clinical settings, this study proposes its use as a primary screening tool for early risk stratification in children infected with RSV. A novel finding of this study is the significant positive correlation between diarrhea and the risk of progression to severe pneumonia among children infected with RSV. Previous studies indicate a bidirectional association between severe RSV pneumonia and bacterial pneumonia, which may explain the occurrence of diarrhea in some children with RSV pneumonia, as bacterial pneumonia is often accompanied by systemic inflammatory responses and gastrointestinal dysfunction (20, 30, 31). We intend to further explore its potential clinical relevance and underlying mechanisms in future investigations.

Our study possesses the following notable strengths. First, we employs real-world clinical data modeling derived from an inpatient pediatric cohort of a regional authoritative medical institution, with a sample size that demonstrates sufficient representativeness for single-center research. Second, this study conducted comparisons across multiple models and ultimately selected the XGBoost model, which exhibited superior prediction accuracy (AUC: 0.949 vs. 0.818) in both training set and test set. The XGBoost model inherently excels in efficiently processing diverse and complex data types, enhancing generalization capabilities, and uncovering intricate interaction relationships among factors. Third, to transcend the limitations of black-box models and enhance interpretability, we employed the SHAP framework to provide robust explanations at both global and local levels. This offers validation for the model's rationality in clinical applications. Thus, this machine learning model with interpretable outputs can effectively bridge complex data analytics and real-world clinical applications, offering potential for personalized risk assessment, prioritized triage, and timely intervention in RSV pneumonia.

This study has several limitations. First, its retrospective design inherently introduces selection and information biases. Although internal validation was conducted, external validation across diverse geographic and demographic populations is required. Second, as a single-center investigation, the study may be subject to sample bias and limited statistical power due to a relatively sample size. Third, despite inclusion of multiple clinically relevant variables, several known predictors of severe RSV pneumonia were not incorporated into the model, highlighting the need for further research. Fourth, the absence of a non-RSV pneumonia control group restricted our ability to compare clinical and laboratory features between RSV positive and RSV negative cases. Finally, this study used real-world data from before and after the COVID-19 pandemic, potentially including pandemic-related systemic shifts. However, the analysis focused on long-term trends and composite scenarios without year or seasonal stratification. Thus, confounding factors from seasonality or pandemic disruptions may not be fully addressed. Future studies should include such controls to enable direct comparative analyses. Prospective, multi-center investigations are warranted to confirm these findings and evaluate the generalizability and clinical utility of the proposed model across different healthcare settings.

## 5 Conclusion

This study integrates and analyzes clinical data of children with RSV pneumonia using machine learning and the SHAP framework, aiming to improve the accuracy of identifying severe RSV pneumonia cases in pediatric patients. Our findings demonstrated that prolonged fever duration, presence of diarrhea, decreased hemoglobin concentration (HGB), absence of rhinorrhea, age under 3 months(Age<3 m), and elevated neutrophil-to-lymphocyte ratio (NLR) were predictors of severe cases among children with RSV pneumonia. Our study provided a feasible and practical tool for the early identification of children at potential risk of severe RSV pneumonia, facilitating more precise risk stratification and individualized clinical interventions. Furthermore, this study is expected to promote the optimization of individualized prevention and treatment plans for RSV infection.

## Data Availability

The raw data supporting the conclusions of this article will be made available by the authors, without undue reservation.
